# Photoluminescence nonlinearity and picosecond transient absorption in an LYSO:Ce scintillator excited by a 266 nm ultraviolet laser

**DOI:** 10.1039/d1ra00347j

**Published:** 2021-05-10

**Authors:** Kun Wei, Dongwei Hei, Qing Xu, Jun Liu, Quan Guo, Xiufeng Weng, Xinjian Tan, Liang Sheng

**Affiliations:** Department of Engineering Physics, Tsinghua University 100084 Beijing China weik16@mails.tsinghua.edu.cn; Northwest Institute of Nuclear Technology 710024 Xi'an China heidongwei@nint.ac.cn

## Abstract

Lutetium–yttrium oxyorthosilicate doped with cerium (LYSO:Ce) is a widely used scintillator, and the study of its nonlinear behavior under high excitation density is very significant owing to its direct influence on radiation measurements. Using a 266 nm ultraviolet laser to excite an LYSO:Ce crystal, the relationship between the photoluminescence (PL) light yield and excitation density was studied by *Z* scan experiments. The excitation threshold of the LYSO:Ce was obtained, which is about 2.3 J cm^−3^. Picosecond transient absorption of LYSO:Ce at 800 nm was obtained and used to analyze the dynamic process of carriers. The physical mechanism behind the nonlinearity was discussed and analyzed using the Förster dipole–dipole interaction model, and the interaction characteristic radius was obtained by fitting. This work can help us understand the nonlinearity phenomenon in scintillators and provide references for related radiation detection applications.

## Abstract 2

PL nonlinearity of LYSO:Ce studied by a UV laser at high excitation density, and the pump–probe absorption experiment used to look into the possible reasons for the nonlinearity effects occurring at high fluxes.

## Graphical abstract

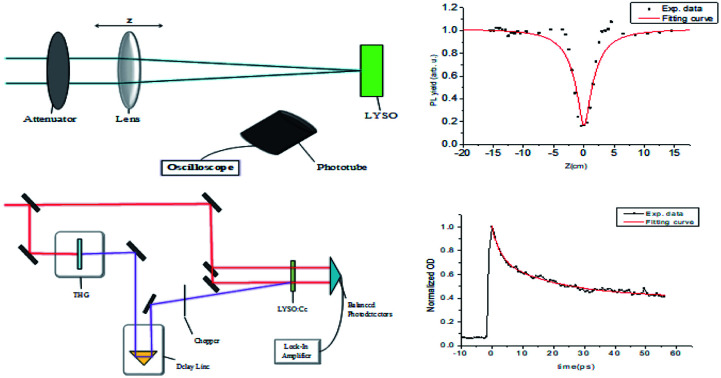

## 1. Introduction

Scintillators are widely used to detect γ/X-rays and charged particles. It is known that the light yield of scintillators is not always completely proportional to the absorbed energy, and the deviation between light yield and absorbed energy is called nonlinearity or nonproportionality.^1–3^ Recently, high peak brightness sources such as free electron lasers (FELs) and laser Compton scattering sources (LCSs) have been developed and applied in different fields. The nonlinearity of scintillators under high excitation density brings new challenges to accurate radiation measurements. Studying this effect is helpful for the accurate diagnosis of source parameters and relevant applications.^4–6^ As an ideal means, ultraviolet laser photoexcitation can be used to quantitatively study the nonlinearity of scintillator crystals.^7–9^ In this paper, the nonlinearity of an LYSO:Ce scintillator excited by an ultraviolet laser at high excitation fluence was studied. In addition, picosecond transient absorption of LYSO:Ce at 800 nm was obtained by pump–probe experiments and used to analyze the dynamic process of carriers.

In 1990, Melcher invented lutetium oxyorthosilicate doped with cerium (LSO:Ce).^10^ It has fast time response, high luminescence yield, strong radiation resistance and no deliquescence, which is particularly suited to high energy physics and nuclear medicine imaging. However, its disadvantage is that lutetium has natural radioactivity, which can cause strong background. The performance of LYSO is similar to that of LSO, but with the addition of yttrium element, the difficulty of crystal growth and manufacturing process is reduced. Therefore, it is considered as one of the inorganic scintillation materials with best comprehensive performance, which can be widely used in different application scenarios. This work can help us to understand the properties of LYSO crystal and guide related application, such as high-energy physics experiments and improved medical imaging devices.

## 2. Experiments

**Fig. 1:**
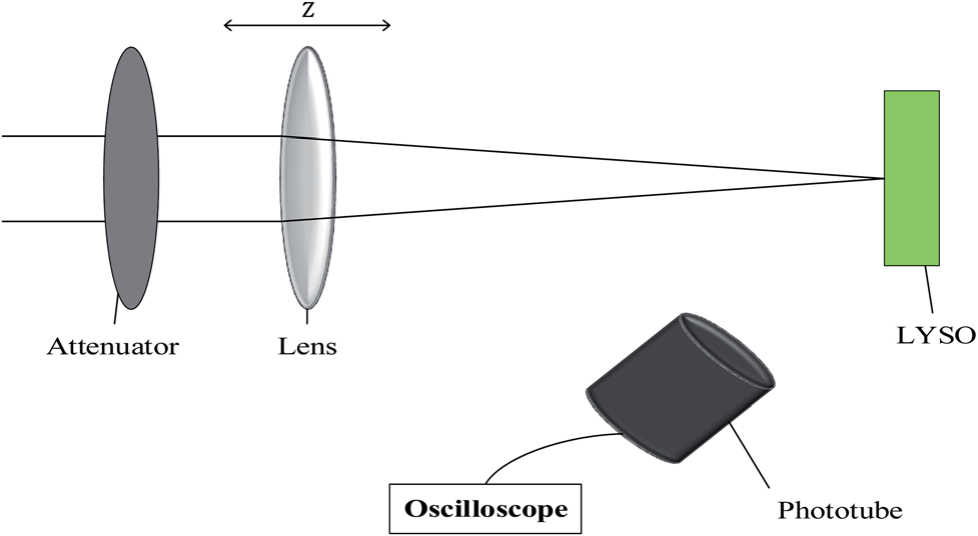
Schematic diagram of *Z* scan experiment settings.

The experiment was set as shown in the Fig. 1. The laser used in the experiment was a 800 nm Ti:sapphire laser. After third harmonic generation and longitudinal shaping, a 266 nm laser with pulse width 10 ps and repetition rate 10 Hz was obtained as the excitation source. The focusing lens (*f* = 300 mm) was placed on an optical translation stage, and the LYSO crystal was fixed perpendicularly to the direction of the laser beam. The photoluminescence (PL) light pulses were detected and recorded by a phototube and a digital oscilloscope. The phototube we used was a GD40 with rise time about 0.5 ns and linear current about 10 A. And the laser power and spot size were obtained by a laser power meter and a M2 beam quality analyzer (Thorlabs BC106-UV). The laser energy was about 160.4 μJ, the diameter of the focal spot was about 80 μm, and the M2 was about 4.24. Then, interband laser *Z*-scan experiment technology was used, which is often used to measure the optical nonlinear characteristics of crystals. By changing the distance between the sample and the laser beam focus, the number of photons on the crystal was same but the fluence was different because the size of the laser spot on the crystal was different. Therefore, the PL decay curves of LYSO:Ce under different fluence were obtained. Some of the experimental results have been presented in ref. 11. The LYSO:Ce crystal sample used in the experiment was 5 cm in diameter and 3 mm in thickness, which was Lu_1.9_Y_0.1_SiO_5_ with 0.5 mol% Ce doping concentration (4 × 10^20^ cm^−3^) and was fabricated by Shanghai Epic Crystal Co., Ltd. (China).

**Fig. 2:**
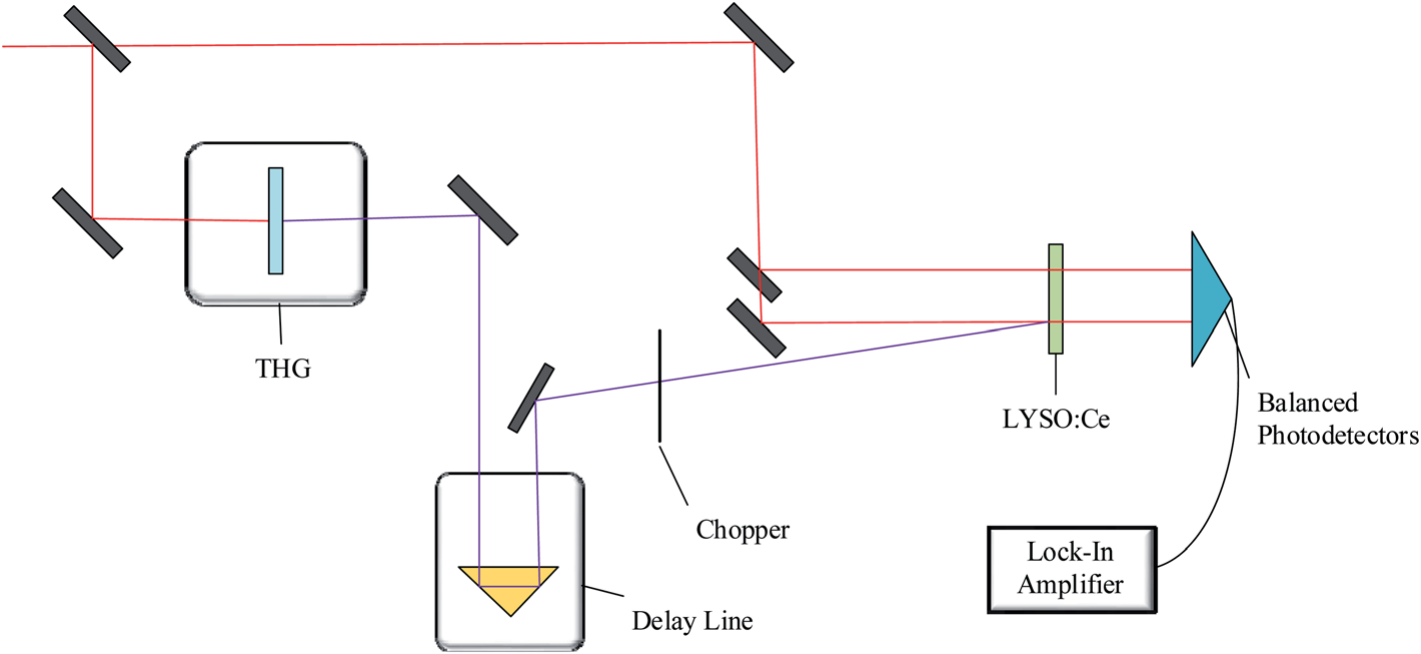
Schematic diagram of pump–probe experiment settings.

In addition, a pump–probe system has been set up to study the dynamic process of carriers in LYSO:Ce excited by 266 nm laser. The optical path diagram are shown in Fig. 2. The 800 nm laser beam was divided into two laser beams by a beam splitter. One of them was used to form 266 nm ultraviolet laser through third harmonic generation and as pump laser to excite sample after through a delay line and a chopper. The other 800 nm laser beam was divided into two beams by a beam splitter again, one beam through the sample unexcited region and the other through the excited region. Then the 800 nm laser beam through the sample were received by the two photocathodes of balanced photodetectors respectively, and the difference signal was input into the lock-in amplifier. The carrier dynamic process can be studied by the change of LYSO:Ce crystal absorption optical density (OD) at 800 nm. The pulse width of 800 nm laser was about 100 fs, so the time resolution of the experiment was less than 200 fs.

## 3. Results

**Fig. 3:**
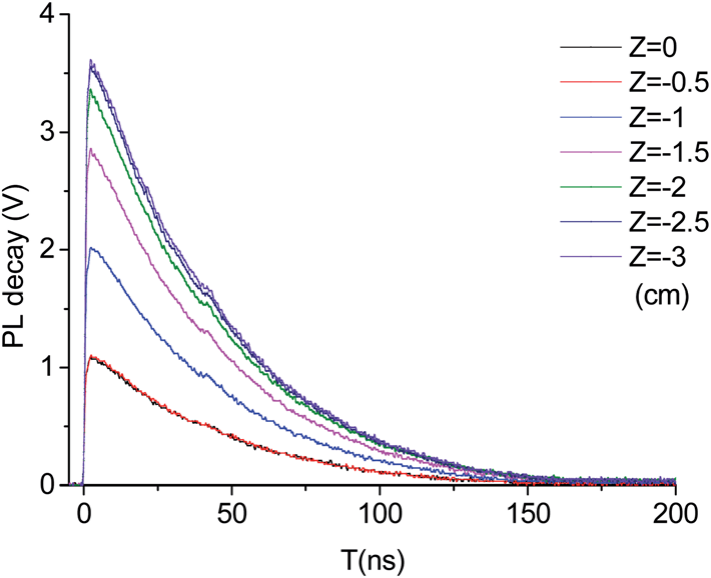
PL decay curves at different distances from focal spot.

**Fig. 4:**
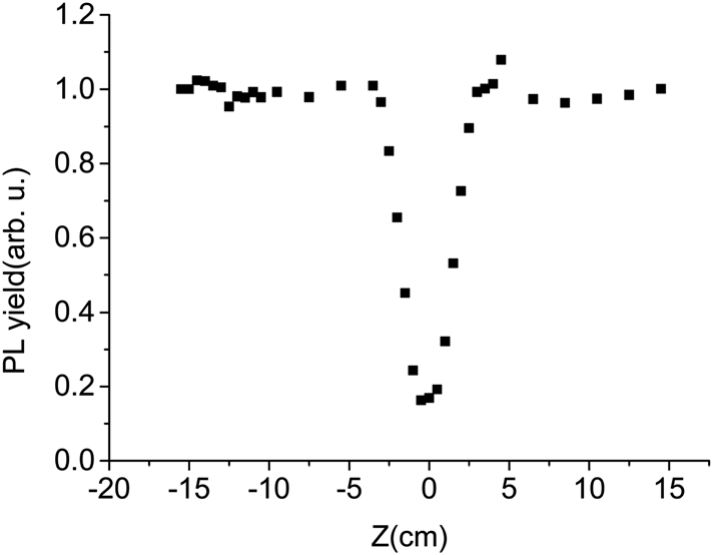
Normalized PL yield at different distances from focal spot.

**Fig. 5:**
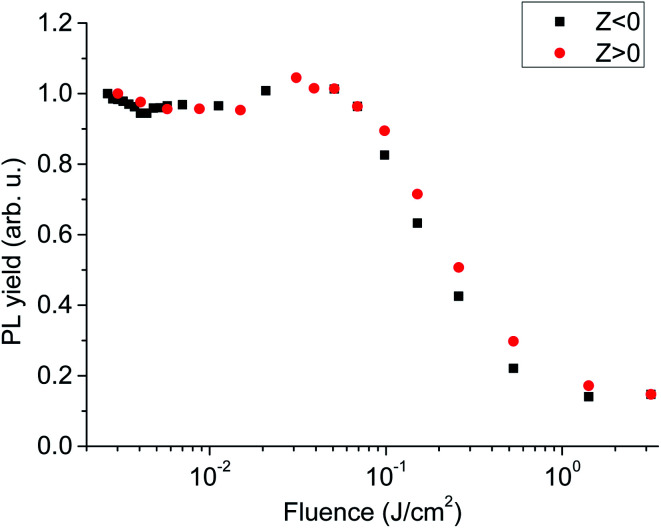
Normalized PL yield with laser fluence.

In the experiment, PL decay curves of LYSO:Ce scintillator excited at different distances from focal spot were obtained, some of which are shown in Fig. 3. We used the intensity of time-integrated PL decay curve as PL yield, and normalized PL yield at different distance from focal spot are shown in Fig. 4. It can be seen from Fig. 4 that the normalized PL yield of LYSO scintillator gradually decreases with the decrease of distance from focal spot, namely the increase of laser fluence. And the relation curves between PL yield and laser fluence were plotted in Fig. 5. The PL yield remains nearly constant from 0 to about 10^−1^ J cm^−2^ and then drops abruptly to about 20% before the fluence reaches 1 J cm^−2^, and some points in Fig. 5 higher than 1 may be from experimental error which can also be seen in Fig. 4.

**Fig. 6:**
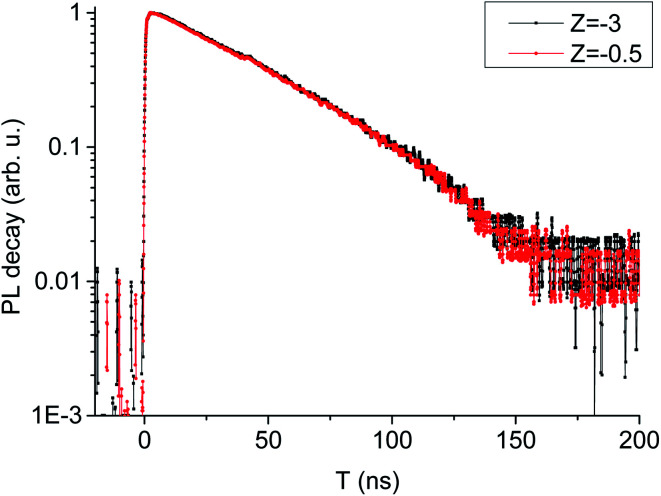
PL decay curves at two different distances.

In order to analyze the effect of excitation density on photoluminescence decay behavior, normalized PL decay curves in two different distance have been plotted in Fig. 6. From Fig. 6, we can see that PL decay behavior is almost unaffected by the excitation density which is decided by the distance between the sample and the laser beam focus (*Z*) in our experiment. It is usually assumed that luminescence in LYSO:Ce is from radiative recombination of the Ce^3+^ state 5d_1_, and the luminescence decay time is determined by the rate of this radiative recombination. Therefore, we believe that the quenching process is faster than the radiation recombination, which may exceed the temporal resolution of the detection system. However, the pump–probe experiment can realize the detection of this ultra-fast process.

In pump–probe experiments, the normalized OD value of LYSO:Ce crystal at 800 nm within about 60 ps after excitation has been obtained. In order to study the time characteristics of carrier dynamics process within picosecond scale, the decay curve was fitted by double exponential function as eqn (1).

1 *y* = *A*_1_ exp(−*x*/*t*_1_) + *A*_2_(−*x*/*t*_2_) + *y*_0_

**Fig. 7:**
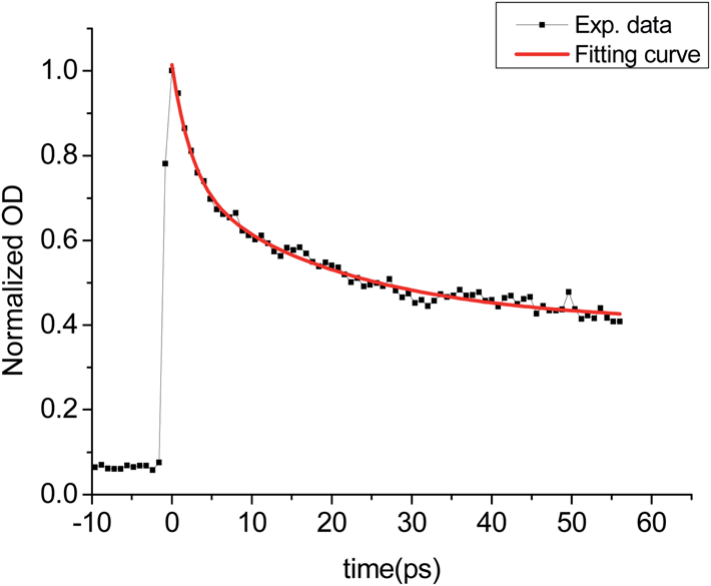
OD decay curve of LYSO:Ce crystal at 800 nm.

The experimental results and the corresponding fitting results are shown in Fig. 7. The fitting parameters are shown in Table 1, from which we can see that there are fast process (∼2 ps) and slow process (∼20 ps) in LYSO:Ce excited by 266 nm laser.

**Table 1:** Fitting parameters of the double exponential function given in eqn (1) for LYSO:Ce scintillator

Fitting parameters	*y*_0_	*A*_1_	*t*_1_ (ps)	*A*_2_	*t*_2_ (ps)
LYSO	0.404	0.330	20.8	0.280	2.56

**Fig. 8:**
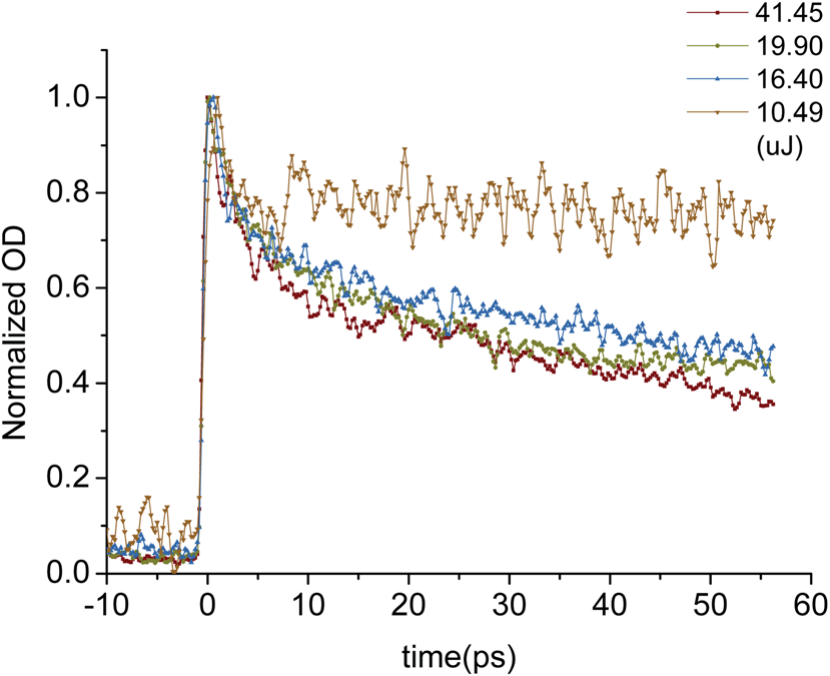
Normalized OD decay curves of LYSO:Ce excited by different energy laser pulses.

In order to study the effect of excitation density on carrier dynamics process, the OD decay curves of LYSO crystal at 800 nm excited by different intensity laser pulses were obtained. The normalized OD decay curves are shown in Fig. 8. It can be seen that the decay of OD curves is affected by the laser pulse energy namely excitation density. The OD curve is basically consistent within a certain range, but when the excitation density is low, the fast process is reduced while the slow process is basically constant. From this, we can draw one conclusion that the fast process comes from high density carriers, which will be weakened as the decrease of the carrier concentration due to low excitation density.

## 4. Discussion

**Fig. 9:**
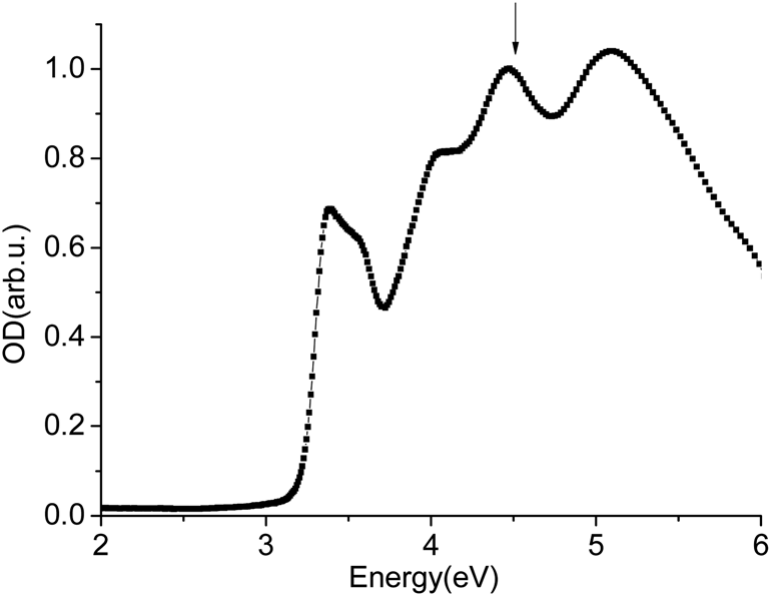
Absorption spectra of LYSO(Ce).

It is necessary to know the luminescence mechanism of scintillators to study scintillator nonlinearity.^12–14^ For different scintillators, the luminescence mechanism is different. According to previous study, the luminescence center of LYSO:Ce scintillator is Ce^3+^ ion, which is used as activators. In our experiments, the excitation laser photon energy is 4.66 eV, which is lower than the energy gap 6.2 eV of LYSO.^15^ However, from the absorption spectra of LYSO(Ce) shown in Fig. 9, the laser photo can excite the Ce^3+^ from the ground state to the third excited state. And there will existence a Frenkel exciton based on excited state of Ce^3+^ with 4f hole component and 5d electron component.^16^ And in pump–probe experiments, pump photon is used to excite the Ce^3+^, and the probe photon can be absorbed by the excited Ce^3+^ to higher excited state.^23^

The decrease of PL yield under high excitation density shown in Fig. 4 and 5 means that quenching process occurs. The results of picosecond transient absorption of LYSO:Ce at 800 nm by means of pump–probe experiments shows there is fast quenching process which is more obvious at high excitation density. In our experiments, high density Frenkel excitons are created by photo-excited, whose quenching process can be explained by Förster dipole–dipole interaction.^17–19^ Therefore, this allows us to use an approach developed for excitonic emission given by^20–22^ to obtain a rough estimate of the Ce^3+^ activators emission under high excitation density, from which we can obtain the relationship between the total PL yield *I*_tot_ with laser pulse energy *I*_0_ as eqn (2).2
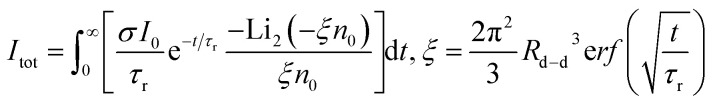


In eqn (2), *I*_0_ is the number of photons in a laser pulse which is the same in our experiments, *σ* is the excitation efficiency which stands for the number of excitons created by one photon, *τ*_r_ is the radiation decay time, 
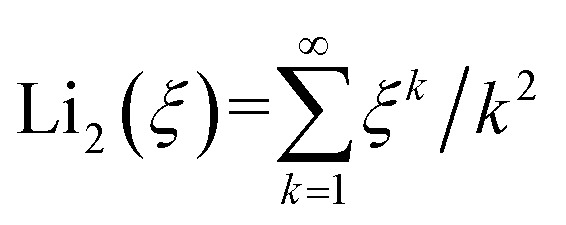
 is dilogarthmic function, *R*_d–d_ is characteristic radius of Förster interaction, and *n*_0_ = *σI*_0_*α*/π*a*^*2*^ is the initial exciton density where *a* is the Gauss radius of laser beam and *α* is the absorption coefficient of LYSO:Ce crystal at 266 nm.

**Fig. 10:**
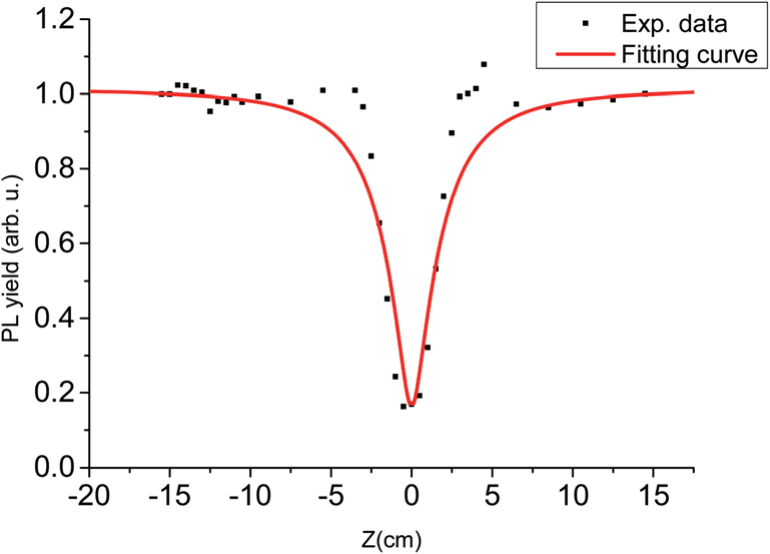
Fit of PL efficiency with laser fluence.

The above model was used to fit the data obtained from our experiments, and the relation curves of PL yield with laser pulse fluence (*Z*) was fitted in Fig. 10. In Fig. 10, we used eqn (2) to fit the relationship between PL yield *I*_tot_ and laser fluence *F* = *I*_0_/π*a*^*2*^ = *n*_0_/*σα*, where the fitting parameters obtained is the characteristic radius *R*_d–d_ = 5.2 nm, and the value of *R*_d–d_ is approximate with that obtained from other scintillator materials in ref. 20–22. From the fitting result, we can see that the model used here roughly agrees with the experiment data. However, there is also the possibility of the Auger process that is well-known interaction for high-density carriers in semiconductors, which may be one of the reasons for the difference between the fitting curve and the experimental data.

From Fig. 4 and 5, we can see that the PL luminous efficiency remains constant in a certain range of low excitation fluence, so we define the excitation density corresponding to a 10% reduction in luminous efficiency as the nonlinear threshold of the scintillator. The laser fluence threshold *F* and the deposition energy density threshold *D* corresponding to 10% PL efficiency nonlinearity of the scintillator LYSO:Ce have been shown in Table 2, where *D* = *Fα*, and *α* is the measured absorption coefficient of LYSO:Ce crystal at 266 nm. From Table 2, we can see that the nonlinearity of LYSO:Ce crystal exceeds 10% when the energy density threshold is above 2.3 J cm^−3^. However, the accuracy in determination of the real excitation energy density at optical excitation is really a problem, so the error obtained here may be more than 10%.

**Table 2:** Laser fluence *F* and energy density threshold *D* corresponding to 10% nonlinearity of LYSO(Ce)

Scintillator	*F* (J cm^−2^)	*α* (cm^−1^)	*D* (J cm^−3^)
LYSO(Ce)	0.024	95 ± 5	2.3

## 5. Conclusion

We studied the relationship between photoluminescence yield of LYSO:Ce and excitation fluence with 266 nm ultraviolet laser excitation by *Z* scan experiments. The experiment results show that the nonlinear response of LYSO:Ce crystal increases with laser excitation fluence. When the deposition energy density is greater than 2.3 J cm^−3^, the PL yield nonlinearity of LYSO:Ce crystal exceeds 10%. Picosecond transient absorption of LYSO:Ce at 800 nm by means of pump–probe experiments has been used to analyze dynamic process of carriers. And the nonlinearity can been explained and calculated by Förster interaction at high excitation density. The radiative recombination process in a similar way after both radiation and optical excitation in LYSO:Ce.^23^ Therefore, the experimental results of optical excitation can also be used for reference in the case of radiation excitation. This work provides a reference for related radiation detection applications based on LYSO:Ce crystal to avoid exceeding the energy threshold, and it is also helpful for us to understand nonlinearity of different scintillators. Further experiments will be done with different types of scintillators, and the physical mechanisms behind the phenomenon worth more in-depth research.

## Conflicts of interest

There are no conflicts of interest to declare.

## Supplementary Material

## References

[cit1] Moses W. W. (2008). Scintillator Non-Proportionality: Present Understanding and Future Challenges. IEEE Transactions on Nuclear Science.

[cit2] Khodyuk I. V. (2012). Trends and Patterns of Scintillator Nonproportionality. IEEE Transactions on Nuclear Science.

[cit3] Moses W. W., Bizarri G. A., Williams R. T. (2012). *et al.*, The Origins of Scintillator Non-Proportionality. IEEE Transactions on Nuclear Science.

[cit4] Kirm M. (2005). Influence of excitation density on luminescence decay in Y_3_Al_5_O_12_:Ce and BaF_2_ radiation in VUV. Phys. Status Solidi C.

[cit5] Vielhauer S., Babin V., De Grazia M. (2008). *et al.*, Self-quenching effects of excitons in CaWO4 under high density XUV free electron laser excitation. Phys. Solid State.

[cit6] Krzywinski J. (2017). Saturation of a Ce:Y_3_Al_5_O_12_ scintillator response to ultra-short pulses of extreme ultraviolet soft X-ray and X-ray laser radiation. Opt. Mater. Express.

[cit7] Laasner R. (2013). *et al.*, Band tail absorption saturation in CdWO_4_ with 100 fs laser pulses. J. Phys.: Condens. Matter.

[cit8] Grim J. Q., Ucer K. B. (2013). *et al.*, Nonlinear quenching of densely excited states in wide-gap solids. Phys. Rev. B: Condens. Matter Mater. Phys..

[cit9] Grim J. Q., Li Q., Ucer K. B. (2011). *et al.*, Experiments on high excitation density, quenching, and radiative kinetics in CsI:Tl scintillator. Nucl. Instrum. Methods Phys. Res., Sect. A.

[cit10] Melcher C. L., Schweitzer J. S. (1992). A promising new scintillator: cerium-doped lutetium oxyorthosilicate. Nucl. Instrum. Methods Phys. Res., Sect. A.

[cit11] Wei K. , Hei D., Liu J., Weng X., Tan X. and Sun B., Light Yield Nonlinearity of LSO Crystal Excited by Picosecond Ultraviolet Laser, in Laser Congress 2019 (ASSL, LAC, LS&C), OSA Technical Digest, Optical Society of America, 2019, paper JM5A.44

[cit12] Vasil'ev A. N. , Microtheory of scintillation in crystalline materials, ed. M. Korzhik and A. Gektin, 2017, pp. 3–34

[cit13] Schleife A. , Zhang X. and Li Q., *et al.*, Excitons in scintillator materials: optical properties and electron-energy loss spectra of NaI, LaBr_3_, BaI_2_, and SrI_2_, 2016, 32(1), 1 8

[cit14] Korzhik M. , Tamulaitis G. and Vasil'ev A., Physics of Fast Processes in Scintillators, Springer, 2020, ISBN 978-3-030-21965-9

[cit15] Auffray E. (2018). *et al.*, Excitation Transfer Engineering in Ce-Doped Oxide Crystalline Scintillators by Codoping with Alkali-Earth Ions. Phys. Status Solidi A.

[cit16] Belsky A. N. (1999). *et al.*, Energy transfer in inorganic scintillators. Radiat. Eff. Defects Solids.

[cit17] Förster T. (1948). Zwischenmolekulare Energiewanderung und Fluoreszenz. Ann. Phys..

[cit18] Clegg R. M., Herman B. (1995). Fluorescence resonance energy transfer. Methods Enzymol..

[cit19] Wu P. G., Brand L. (1994). Resonance Energy Transfer: Methods and Applications. Anal. Biochem..

[cit20] Kirm M. (2009). *et al.*, Exciton–exciton interactions in CdWO_4_ irradiated by intense femtosecond vacuum ultraviolet pulses. Phys. Rev. B: Condens. Matter Mater. Phys..

[cit21] Nagirnyi V., Dolgov S., Grigonis R. (2010). *et al.*, Exciton–exciton interaction in CdWO_4_ under resonant excitation by intense femtosecond laser pulses. IEEE Transactions on Nuclear Science.

[cit22] Markov S. (2012). *et al.*, Modelling of decay kinetics of self-trapped exciton luminescence in CdWO_4_ under femtosecond laser excitation in absorption saturation conditions. Cent. Eur. J. Phys..

[cit23] Tamulaitis G., Aufffray E. (2020). *et al.*, Improvement of the timing properties of Ce-doped oxyorthosilicate LYSO scintillating crystals. J. Phys. Chem. Solids.

